# Vitamin B12 deficiency in a pediatric patient with gastric obstruction and jejunal feeding dependence: A case report

**DOI:** 10.1002/jpr3.70117

**Published:** 2025-12-09

**Authors:** Angela H. Nguyen, Dorsey Bass, Rachel E. Herdes

**Affiliations:** ^1^ Department of Pediatrics Stanford Children's Health Stanford California USA; ^2^ Department of Pediatrics, Division of Pediatric Gastroenterology, Hepatology, and Nutrition Stanford Children's Health Stanford California USA

**Keywords:** congenital intestinal atresia, enterohepatic circulation, intrinsic factor malabsorption

## Abstract

Pediatric B12 deficiency is most caused by insufficient dietary intake, malabsorption or autoimmune gastritis. We present a unique case of B12 deficiency in a pediatric patient with complex gastrointestinal anatomy and jejunal nutritional dependence nearly two decades after unsuccessful surgical intervention. High‐dose enteral B12 supplementation via jejunostomy tube resulted in rapid resolution of the patient's neuromuscular manifestations of vitamin B12 deficiency, highlighting the importance of considering nutritional deficiencies in patients reliant on jejunal enteral nutrition and anatomic differences.

## 1 INTRODUCTION

Vitamin B12 (cobalamin) is a water‐soluble vitamin essential in several physiologic processes, including deoxyribonucleic acid (DNA) synthesis, red blood cell (RBC) development and maturation, and nervous system function. It is absorbed both by passive diffusion and active processes. The recommended daily intake (RDI) per the Institute of Medicine (IOM) and National Institute of Health (NIH) for the United States and Canada is 2.4 mcg per day for adults over the age of 13 (Table 1).^1^

**Table 1:** RDA of vitamin B12 per NIH guidelines.

RDA of vitamin B12	
Age	RDA (micrograms per day)
0–6 months	0.4
7–12 months	0.5
1–3 years	0.9
4–8 years	1.2
9–13 years	1.8
>14 years	2.4

*Note*: This table summarizes the RDA of Vitamin B12 categorized by age groups, as described by the guidelines set forth by the National Institute of Health.

Abbreviations: NIH, National Institute of Health; RDA, recommended daily intake.

Vitamin B12 is initially ingested and released from animal protein by the low pH of the stomach. Parietal cells in the stomach secrete hydrochloric acid and intrinsic factor (IF). In an acidic environment, vitamin B12 binds to haptocorrin and the vitamin B12‐haptocorrin complex is transported to the duodenum, whose increased pH facilitates vitamin B12 to be released from the complex and bind to IF, forming the vitamin B12‐IF complex. The vitamin B12‐IF complex is absorbed through a calcium‐dependent mechanism at the surface of enterocytes, most significantly in the terminal ileum. Within the enterocyte, vitamin B12 is released from IF and is released from the cell via lysozymes back into the portal circulation. This enterohepatic circulation allows vitamin B12 to preserve body stores in the absence of malabsorption.^2^ Once vitamin B12‐IF receptors have been saturated, 1%–2% of enteral vitamin B12 can be absorbed via passive diffusion throughout the gastrointestinal (GI) tract.^2^

Vitamin B12 deficiency is a nutritional deficiency most often associated with low nutritional intake, malabsorption, inborn errors of vitamin B12 transport, or autoimmune processes, such as pernicious anemia.^3^ It has also been identified in patients who undergo metabolic and bariatric surgery (MBS) due to loss of functional parietal cells. These patients subsequently produce less IF, therefore limiting active absorption of vitamin B12 via vitamin B12‐IF complex receptors in the ileum.^4^ Conditions that can lead to vitamin B12 deficiency include small intestinal bacterial overgrowth (SIBO), inflammatory bowel disease (IBD), or short bowel syndrome (SBS) due to loss or dysfunction of the ileum.^4^

Vitamin B12 deficiency presents with nonspecific manifestations, such as developmental delay, irritability, weakness, and malnutrition. Delay in DNA synthesis due to B12 deficiency in rapidly growing hematopoietic cells can result in macrocytic anemia.^3^ Neurologic changes can occur without hematologic abnormalities, including sensory deficits, loss of deep tendon reflexes (DTR), developmental regression, hypotonia, seizures, dementia, and neuropsychiatric changes.^3^

Treatment of vitamin B12 deficiency can include oral or parenteral supplementation. The standard of treatment for symptomatic vitamin B12 deficiency secondary to dietary insufficiency is initiation with intramuscular vitamin B12 therapy, typically with intramuscular (IM) hydroxocobalamin, followed by oral supplementation once neurologic symptoms have resolved. For those impaired vitamin B12 absorption, such as in SBS or those with prior gastric bypass surgery, treatment is often lifelong IM vitamin B12 therapy.^5^

## 2 CASE REPORT

We present the case of a Hispanic 20‐year‐old male patient with a complex medical history including trisomy 21, tetralogy of Fallot (repaired), and intrauterine‐diagnosed esophageal and duodenal atresia who presented to pediatric GI outpatient clinic with acutely progressive tremors, falls and leg cramps over the course of 2 months.

Our patient was originally seen at an outside institution for the first 2 years of life, where he underwent surgical correction of his congenital heart defects, right colonic interposition, tapering duodenoplasty with Roux‐en‐Y. He was hospitalized for the first 16 months of life, and his prolonged hospital course was complicated by multiple small bowel resections and re‐anastomosis due to bowel obstructions. He presented to our institution at 32 months old with a gastrostomy tube (GT) for venting and a jejunostomy tube (JT) for medications and feeding. Unfortunately, there are no available records to review his detailed anatomy before and after his surgical operations. Per parental report, his last surgical revision took place 1 year before presentation at our institution.

**Figure 1:**
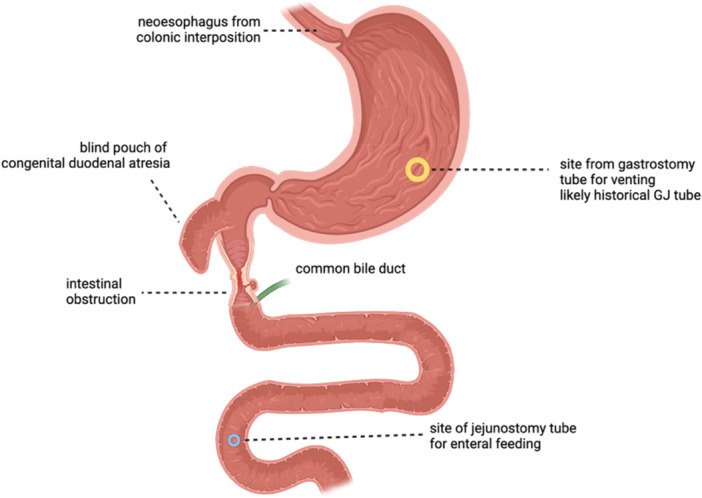
Digital representation of patient anatomy adapted from imaging and procedural reports. This image was created with the assistance of Biorender, an online imaging tool (Nguyen, 2025) https://BioRender.com/1dmy9fc). The writer used a combination of operative reports and radiologic imaging to approximate the patient anatomy in the absence of surgical reports from an outside facility where the surgical repairs occurred. This image shows the site of the gastrostomy tube and jejunostomy tube as well as the approximated site of intestinal obstruction proposed to be causing gastric outlet obstructive symptoms.

Diagnostic esophagogastroduodenoscopy (EGD) at the time of transition to our institution found mild colitis in his esophagus and a stricture at the distal esophagus requiring balloon dilatation to enter the gastrum. The pylorus was patent, and follow‐through into the Roux‐en‐Y area was met with a one limb ending in a blind pouch and another limb that extended at least 10–15 cm. There was no description about the placement of the common biliary tree, but per parental report he had always had nonbilious gastric output. Fluoroscopy with contrast injection in the gastrojejunostomy tube (GJT) in the years following this upper endoscopy showed evidence of obstruction distal to the JT. However, years later, contrast injection of a more distal JT showed adequate filling of the small bowel. Thus, it was shown that our patient was unable to achieve successful repair of his congenital duodenal atresia, with his stomach persistently obstructed and discontinuous from the jejunum (Figure 1). Functionally, his GJT drains gastric contents, including IF, and the JT is used for enteral feeding only. Enterohepatic circulation was likely preserved via ductal bypass at the time of his Roux‐en‐Y procedure allowing him the ability to build some storage of vitamin B12 before his complications with recurrent small bowel obstructions.

At the time of presentation, the patient was afebrile with normal stool output. Radiographs ruled out acute fracture. His weight curve was downtrending even though he was meeting 100% of his caloric goals. He had been having chronic fluctuations in his growth curve in the several years before these new symptoms, so the weight loss did not acutely prompt investigation. However, tremors, falls, and overall muscle weakness were more concerning for neuromuscular manifestations of an underlying metabolic or nutritional deficiency. His jejunal feeding regimen with fortified, hydrolyzed formula supplied 2438 kcal and 10.5 µg of vitamin B12 daily.

Given the broad differential for weakness in the setting of weight loss, screening labs were obtained to evaluate for malabsorption and endocrinopathies (Table 2). A complete blood count showed mildly elevated mean corpuscular volume (MCV) and serum folate level of 39.90 nanograms per milliliter (ng/mL) (normal range 4.80–24.20 ng/mL). Vitamin B12 serum testing found a below detectable level and a significantly elevated methylmalonic acid (MMA) level of 13.46 (normal ≤ 0.40). Homocysteine levels were not obtained upon initial evaluation.

**Table 2:** Lab results at diagnosis and 1 month after enteral cyanocobalamin supplementation.

Lab test	Reference range	Initial value	1 month after enteral cyanocobalamin supplementation 2000 mcg daily
WBC	4.0–11.0 K/μL	**13.9 (H)**	8.7
Hemoglobin	13.5–17.7 g/dL	17	15.8
Hematocrit	40.0%–52.0%	51.1	47.7
Platelet Count	150–400 K/μL	356	307
MCV	82.0–98.0 fL	**99.6 (H)**	96.8
RDW	11.5%–14.5%	14.1	13
RBC	4.40–5.90 MIL/μL	5.13	4.93
MCH	27.0–34.0 pg	33.1	32
MCHC	32.0–36.0 g/dL	33.3	33.1
Fat stain, stool	Negative	Negative	
Elastase‐1, stool	>200 mcg/g	206	
Free T4	0.93–1.70 ng/dL	1.32	1.43
TSH	0.27–4.20 μIU/mL	1.56	**4.24**
Folic acid (folate)	4.80–24.20 ng/mL	**39.90 (H)**	
Vitamin B12 (cobalamin)	232–1245 pg/mL	**<150 (L)**	**3450 (H)**
Methylmalonic acid, quantitative, serum	≤0.40 nmol/mL	**13.46 (H)**	0.31
IgA, serum	68–408 mg/dL	345	
IgA anti TTG antibody	Negative	Negative	
IgA anti TTG level	<8.0 U/mL	1.1	
IgG anti DGP antibody	Negative	Negative	
IgG anti DGP level	<8.0 U/mL	<1.0	
**(H)**: Data are abnormally high			
**(L)**: Data are abnormally low			

Abbreviations: DGP, deaminated gliadin peptide; dL, deciliter; fL, femtoliter; g, gram; IgA, immunoglobulin A; IgG, immunoglobulin G; K, thousand; L, liter; mcg, microgram; MCH, mean corpuscular hemoglobin; MCHC, mean corpuscular hemoglobin concentration; MCV, mean corpuscular volume; mg, milligram; MIL/μL, millions per microliter; mL, milliliter; ng, nanogram; pg, picogram; RBC, red blood cell; RDW, red cell distribution width; T4, thyroxine; TSH, thyroid stimulating hormone; TTG, tissue transglutaminase; U, unit; WBC, white blood count; μIU/mL, micro‐international units per millilitre; μL, microliter.

He was diagnosed with vitamin B12 deficiency and initiated 2000 mcg of enteral cyanocobalamin via the JT once daily, estimating to provide about 20‐40 mcg of daily vitamin B12 via passive diffusion. Follow up 6 weeks after vitamin B12 supplementation found improved serum MMA levels (Table 2), resolution of all neurologic symptoms, and improved growth trajectory.

## 3 DISCUSSION

Our patient's B12 deficiency was likely due to insufficient dietary intake and chronic intestinal obstruction from unsuccessful duodenal atresia repair. Because his stomach is not contiguous with his distal intestinal tract after multiple attempts at re‐anastomosis, IF produced in the stomach cannot be transported to the ileum. While he likely has intact ileum, his small bowel obstruction, as illustrated by the fluoroscopy findings, inhibits the transit of IF and its role in active absorption. Enterohepatic circulation with biliary drainage distal to the obstruction may have sustained levels for some time through passive diffusion, but once depleted could not be restored. Passive absorption would have provided 0.1–0.2 mcg of vitamin B12 daily, significantly below the recommended dietary intake (RDA) as described by the NIH.^1^

There were no records documenting whether the patient had a pre‐ or postampullary duodenal atresia. This distinction would have helped to explain his late presentation of symptoms. Before and between multiple attempts at re‐anastomoses in the years following his initial repair, the patient likely sustained bowel continuity for active B12 absorption and storage to occur. Postampullary duodenal atresia would have maintained enterohepatic circulation and allowed for stored vitamin B12 to be utilized in the years leading up to clinical presentation. If he had preampullary duodenal atresia, ductal bypass would have achieved this function as well. Regardless, the integrity of enterohepatic circulation must have remained intact to explain his delayed presentation of symptoms.

High levels of enteral vitamin B12 supplementation can restore normal serum levels of vitamin B12 even without IF, as both enteral and parenteral supplementation have been shown to adequately treat vitamin B12 deficiency. While we acknowledge that the standard of care for neurologic symptoms of vitamin B12 deficiency prompt initial treatment with intramuscular vitamin B12 in the form of cyanocobalamin or hydroxocobalamin every other week until improvement before maintenance therapy of oral supplementation, our patient exhibited only mild neurologic symptoms that did not warrant a more immediate solution. There has been some evidence in recent systematic reviews that found high‐dose enteral supplementation of 2000 mcg daily of vitamin B12 can be comparable to intramuscular administration of vitamin B12.^5^ This patient subsequently showed rapid resolution of symptoms with high‐dose enteral supplementation and remained on this maintenance dose.

This patient's complex surgical history and ongoing small bowel obstruction raises the risk of developing SIBO. Several factors within SIBO could have also played a role in vitamin B12 deficiency, such as consumption of cobalamin by bacteria, impaired absorption due to competitive binding of bacterial metabolites, or impaired absorption due to mucosal damage.^6^ Since the patient had significant improvement of neuromuscular symptoms and weight gain with vitamin B12 supplementation, treatment for possible SIBO was not considered.

In the case of this patient's unique anatomy, we should consider other possible nutrient deficiencies that may have developed from patients with chronic intestinal obstruction. In the stomach, parietal cells secrete hydrochloric acid, creating an acidic environment that kills some bacteria and facilitates downstream absorption of iron in the jejunum.^7^ Gastric obstruction can limit iron absorption due to an alkaline intestinal milieu, depending more on downstream transporters in the GI tract to maintain normal iron levels.^8^ The human proton‐coupled folate transporter is highly expressed in both the duodenum and proximal jejunum, so folate levels should also be carefully monitored in patients with duodenal atresia.^9^ Blood counts in our patient were reassuring against iron or folate deficiencies, indicating there was sufficient dietary intake via enteral formula. However, for future cases, there is a need to monitor for these nutritional deficiencies.

This case is unique as vitamin B12 deficiency most commonly is thought to arise from insufficient dietary intake, malabsorption, or secondary to gastrectomy resulting in parietal cell bulk and loss of intrinsic factor production. No case reports have attributed vitamin B12 deficiency in a patient with altered anatomy due to gastric losses and chronic intestinal obstruction.

## 4 CONCLUSION

In conclusion, this case highlights the importance of screening for vitamin B12 deficiency and other elemental and vitamin deficiencies in patients with enteral tube feeding dependence or complex gastrointestinal anatomy. This patient's vitamin B12 deficiency likely resulted from impaired intrinsic factor transport and active absorption due to anatomical disruptions. However, enteral vitamin B12 supplementation via the jejunal tube successfully restored serum levels and reversed neurologic symptoms. This case emphasizes the importance of monitoring for nutritional deficiencies in patients with altered gastrointestinal anatomy and function. Early diagnosis and treatment can improve clinical outcomes in such patients.

## CONFLICT OF INTEREST STATEMENT

The authors declare no conflict of interest.

## ETHICS STATEMENT

The authors of this article obtained informed consent from the patient's parent before conducting the review for this report. They were made aware of the intent to publish and agree for the patient's clinical case to be used in this case report.
